# The Enactive Torch: Open-source sensory substitution device for neurophysiological research

**DOI:** 10.1016/j.ohx.2026.e00784

**Published:** 2026-05-22

**Authors:** Stephen Estelle, Lohith Dayantri, Ziwen Meng, Brian Morrissey, Tom Froese

**Affiliations:** Embodied Cognitive Science Unit, Okinawa Institute of Science and Technology, 1919-1 Tancha, Onna, Kunigami District, Okinawa, 904-0495, Japan

**Keywords:** Sensory substitution device, Agency, Neurophysiology, Haptic, Enactive Torch

## Abstract

We present the updated Enactive Torch (ET), an open-source, multimodal sensory substitution device engineered to facilitate synchronized haptic stimulation and neurophysiological data acquisition, particularly electroencephalography (EEG). Designed for modularity and accessibility, the ET converts distance measurements from a LiDAR sensor into vibratory feedback via internal and external actuators, enabling embodied perception studies across diverse research contexts. The system includes a Teensy 4.0 microcontroller, a real-time trigger interface, and a user-programmable C++ framework that supports multiple operational modes (e.g., auto, manual, binary). Key hardware components are validated through latency testing and vibrational performance characterization, confirming sub-25 ms system responsiveness. The ET also integrates seamlessly with EEG systems via synchronized triggers, allowing for precise event marking in experimental paradigms. All firmware, schematics, 3D models, and build instructions are publicly available to support reproducibility and community-driven development. This platform enables researchers to explore novel paradigms in enactive cognition, sensory augmentation, and human-machine interaction with minimal engineering overhead

## Graphical abstract

**Figure:**
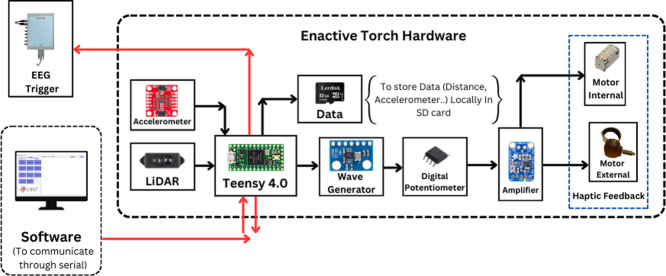

## Specifications table

**Table:** 

Hardware name	Enactive Torch

Subject area	• Neuroscience • Educational Tools and Open Source Alternatives to Existing Infrastructure

Hardware type	• Measuring physical properties and in-lab sensors • Electrical engineering and computer science • Mechanical engineering and materials science

Closest commercial analog	No direct commercial analog is available. While some sensory substitution tools exist (e.g., BrainPort, vOICe), they differ significantly in modality, integration capabilities, and open-source accessibility. The Enactive Torch is uniquely characterized by its real-time EEG synchronization, haptic feedback customization, and open-source design.

Open source license	Creative Commons Attribution License

Cost of hardware	Approximately $500 USD

Source file repository	https://osf.io/vrsya/overview

## 1 Hardware in context

Sensory substitution devices (SSDs) have been the focus of sustained research aimed at restoring or enhancing perceptual abilities in individuals with sensory impairments. By remapping one modality (e.g., vision, sound, or touch) to an alternative, unimpaired modality, SSDs offer a compensatory pathway through which users can perceive environmental stimuli. These devices are currently used as investigative tools in numerous studies examining brain activity, cognitive effort, and the activation of specific neural substrates in healthy and clinical populations. For example, SSDs have been used to explore long-term neuroplastic adaptations using functional MRI (fMRI) following extended training [1], [2], as well as to assess slow, time-scale physiological changes during experimental sessions.

In many of these investigations, SSD data collection occurs before or after training sessions [3], bypassing the need for precise temporal alignment with simultaneously recorded physiological signals. However, a growing body of research now requires real-time synchronization between SSD outputs and neurophysiological measurements—such as electroencephalography (EEG)—to capture events of interest as they unfold during active device use. Yet, most studies limit synchronization to the start and end points of the task [4], [5] or rely on manual insertion of event markers during post-processing [6]. In other cases, although device–physiology synchronization is achieved, it often omits active exploration and fails to mark salient behavioral or cognitive events in real time [7].

To address this methodological shortcoming, we introduce the latest version of the Enactive Torch (ET), a versatile SSD designed to interface seamlessly with concurrent data acquisition systems. The ET enables synchronized recording of behavioral and physiological signals during active exploration. Previous iterations of the ET [8], [9] provided external haptic feedback proportional to real-time distance measurements but offered limited control over the haptic output waveform, defaulting to a pulse-width modulated (PWM) square wave. Additionally, data from built-in accelerometers and haptic sensors were transmitted only after trial completion via Bluetooth, precluding real-time synchronization. Earlier versions also used interchangeable distance sensors limited to either short- or long-range detection, making simultaneous multi-range acquisition impossible.

The current ET model rectifies these constraints while introducing improvements discovered through iterative design. First, we have replaced Bluetooth transmission with a wired triggering interface that synchronizes critical events in real time, enhancing compatibility with EEG and other physiological recording systems. Second, the upgraded distance sensor now captures both short and long range data using a single sensor, enabling greater experimental flexibility. Third, a dedicated waveform generator produces a sine wave output for haptic feedback, overcoming the previous reliance on square wave PWM signals. Furthermore, an internal motor has been added to complement the external actuator, broadening the scope of potential experimental tasks. Lastly, researchers can now store real-time data to an SD card and adjust vibration parameters (e.g., voltage, frequency) via digital potentiometers and wave signal generators, thus enabling a more sophisticated relationship between distance measurements and haptic outputs.

### 1.1 Utilization

The ET sensory substitution device has been adopted by multiple laboratories outside of Japan (Table 1), with a variety of research groups in fields such as embodied cognitive science and psychology incorporating the ET into their experimental frameworks.

At the University of Cincinnati, researchers evaluated participants’ abilities to judge pass-through affordances using vision, a conventional rod, and the ET—investigating whether visual input remains the most reliable modality [10]. At the Autonomous University of Madrid, scholars examined the use of the ET for navigating around obstacles [11]. Investigators at the National Autonomous University of Mexico (UNAM) explored whether the ET could foster active engagement and enhance perceptual skills through embodied interaction [9]. At the University of Central Florida, the focus was on understanding how person-plus-tool systems might extend or augment cognitive processes [12]. Meanwhile, researchers at Illinois State University sought to determine which parameters users calibrate while engaging in goal-directed tasks with the ET [13].

As the ET user community continues to expand, new research questions and methodological refinements emerge. Ongoing improvements to the ET device and its foundational methodologies have the potential to advance experimental paradigms and yield novel insights into the principles and applications of sensory substitution and cognitive augmentation.

**Table 1:** Summary of published studies utilizing the Enactive Torch. Each study employed the device to investigate diverse research questions across perceptual, cognitive, and sensorimotor domains.

University	Developer	Research question
University of Sussex	Grespan et al. 2008 [14]	To understand how enactive perception, involving bodily interactions with the environment, facilitates the construction of spatial awareness
University of Limerick	McGann et al. 2011 [15]	This study examines whether using the ET leads to changes in body image
University of Sussex	Froese et al. 2012 [8]	It aims to explore how the ET enables a novel sensory modality and how it can provide insights into embodied cognition
University of Cincinnati	Favela et al. 2018 [10]	How does the ability to judge pass-through affordances change using vision, rod, and the ET
National Autonomous University of Mexico (UNAM)	González-Grandón et al. 2018 [9]	Does the ET promote active engagement and improve perceptual skills through embodied interaction
Autonomous University of Madrid	Lobo et al. 2019 [11]	How efficient is the ET for participants to solve navigational tasks
University of Central Florida	Favela et al. 2021 [12]	Do person-plus-tool systems effectively extend or augment cognitive processes
Illinois State University	Duffrin et al. 2024 [13]	What parameters are calibrated by users during goal-directed tasks performed with the ET

## 2 Hardware description

The ET is powered by a Teensy 4.0 microcontroller and integrates 14 distinct components to ensure full operational functionality. The device’s hardware is enclosed in a custom, ergonomically designed chassis produced using 3D printing techniques. To facilitate reproducibility and encourage community-based advancements, all relevant documentation—including detailed assembly instructions, bill of material, C++ source code, and 3D design files—are freely available online.

Replicating the ET requires a skill set encompassing fundamental electrical engineering and fabrication competencies. In particular, proficiency in circuit assembly is essential to ensure compact and reliable connections among the various components. Soldering and wire-splicing techniques are critical to achieving secure electrical joints, while familiarity with uploading firmware and configuring microcontrollers (e.g., using the Arduino IDE or equivalent toolchains) is necessary to program the Teensy 4.0 device. Additionally, expertise in 3D printing and materials selection will facilitate in-house production of the device’s enclosure. For laboratories without direct 3D printing capabilities, outsourcing fabrication to a third-party manufacturer is an alternative option.

In summary, constructing the ET requires a technical background in electronics, programming, and fabrication. Researchers equipped with these competencies can reproduce the ET to support a wide range of sensory substitution experiments, furthering the development of hyperscanning methodologies and interdisciplinary research initiatives.

### 2.1 Hardware

**Fig. 1:**
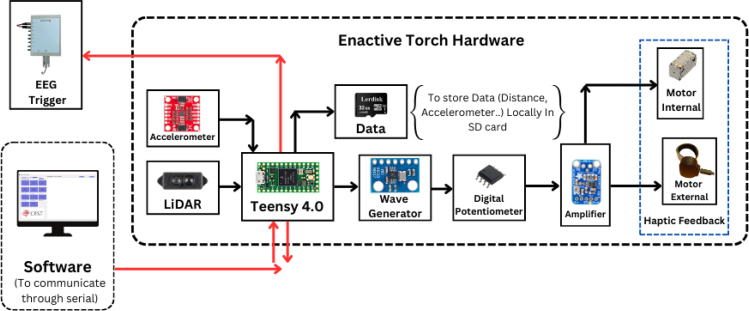
Information flow within the Enactive Torch system. The device is governed by a Teensy 4.0 microcontroller, which acquires distance measurements, user-input signals, and accelerometer data. Processed signals — including trigger commands, real-time sensor data, and generated wave frequencies — are output to drive the haptic feedback actuators.

The ET’s functionality is governed by a Teensy 4.0 development board (Fig. 1), selected for its compact form factor and robust specifications, including 1984 KB of flash memory, 1024 KB of RAM, and a 600 MHz CPU speed. At the front end of the ET, a 5 V, 6 mm, 650 nm red-dot laser pointer provides a visual reference, allowing both the user and observers to approximate the ET’s pointing direction. For distance sensing, the TFMini-S Micro LiDAR Module (SEN-16977) was employed, offering a detection range spanning 0.1 to 12 m.

A waveform generator (AD9833) is integrated to produce a stable sine wave signal, addressing the default square-wave output of the Teensy, which is not optimal for precise haptic feedback. The sine wave output is routed through a digital potentiometer (MCP4151-503E/SN), enabling adjustable amplitude control. This configuration grants experimenters enhanced flexibility to tailor the haptic output to specific experimental requirements. By modulating the PWM values between 0 and 255, researchers can fine-tune the device’s vibrational characteristics.

**Fig. 2:**
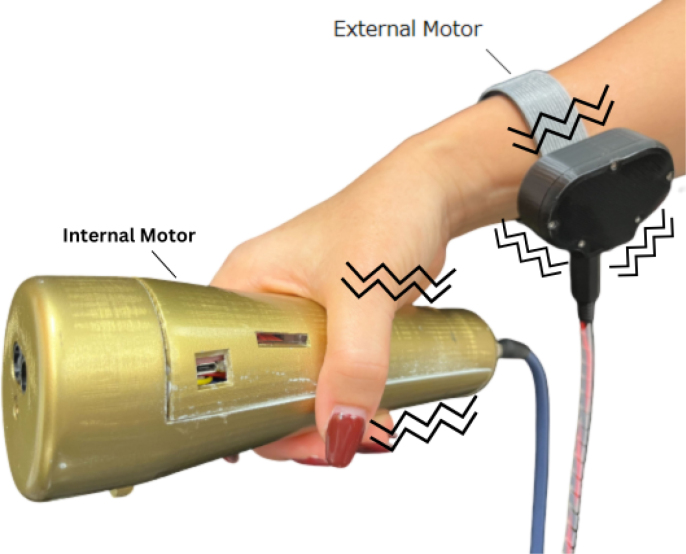
The haptic stimulus is applied to the inner surface of the user’s palm while holding the device, or alternatively to any body location connected to the external vibration motor module.

As the sine wave is inherently weaker than a square wave, an Adafruit Mono 2.5 W Class D audio amplifier (PAM8302) ensures the signal is sufficiently amplified. From the amplifier, the signal passes through a 2-way switch (8SS1011-Z), which can be manually toggled to direct the haptic feedback internally (within the ET’s housing) or externally. A linear resonant actuator (AFT14A903 A) then converts the electrical signal into mechanical vibration, delivering haptic feedback either directly to the user’s hand (internal actuation) or to an external mounting location for more flexible application (Fig. 2).

In addition to haptic functionality, the Teensy board interfaces with a 9-degree-of-freedom accelerometer (SEN-15335) to capture roll, pitch, yaw, orientation, and acceleration data. These motion parameters, combined with distance measurements from the LiDAR and relevant software outputs, are recorded onto a microSD card using the Teensy Micro SD Card Adapter. Power is supplied via two separate 3.7 V, 1000 mAh LiPo batteries (DTP603450), each connected to its own Sparkfun LiPo Charger/Booster (PRT-14411), enabling recharging and stable 5 V, 1 A outputs for powering the Teensy and peripheral components.

The ET’s outer casing and the external motor enclosure are fabricated from 3201PA-F Nylon using Selective Laser Sintering (SLS), resulting in a robust yet lightweight and ergonomically shaped housing. A transparent resin window, produced through Stereolithography (SLA) translucent 8001 Resin 3D printing, is bolted onto the ET’s side to provide a view of the internal LED indicator that conveys battery status during charging. Finally, a 20-pin cable is attached to the ET’s rear panel, organizing all connections between the ET, the host computer, and the external motor assembly.

### 2.2 Circuitry

The ET’s circuitry is designed with the future goal of producing easily replicable printed circuit boards (PCBs), thereby facilitating broader dissemination and adoption of the device by researchers lacking extensive electrical engineering expertise.

Both the internal and external actuators are programmed to operate exclusively between 60 Hz and 145 Hz. Although these actuators possess resonant frequencies near 320 Hz and 160 Hz, these higher frequencies are intentionally avoided to reduce extraneous noise. By selecting a frequency range just below the initial resonance point, the ET provides a more stable and comfortable user experience.

**Fig. 3:**
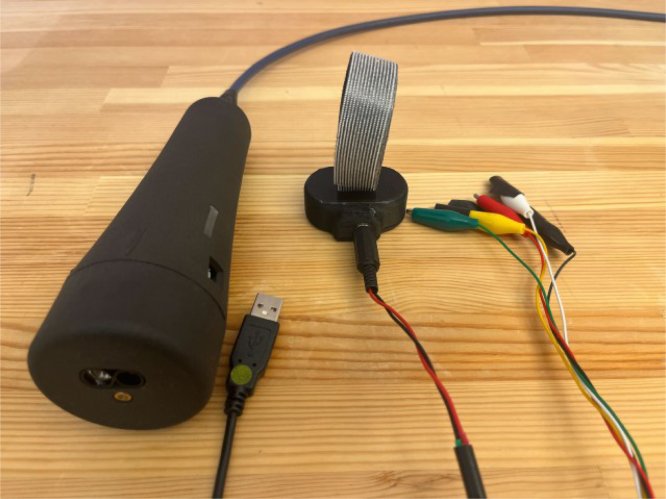
The Enactive Torch and its external modules. Shown components include the computer interface modules, trigger connections, and the external vibration motor.

In addition, the ET is capable of delivering four distinct trigger signals for synchronization with EEG recording software (Fig. 3). These four signals, each represented in binary (HIGH/LOW), can be combined to produce up to 16 unique triggers (e.g., 1011, 0110, 0001), of which 15 are utilized in practice. The default state is represented by all signals set to LOW (0000), ensuring a stable baseline. Fig. 3 illustrates five connection pins — green, yellow, red, white, and black — corresponding to the four binary triggers plus a ground reference.

### 2.3 Software

The ET’s core functionality is governed by C++ code developed within the Arduino IDE environment, utilizing specialized libraries such as TeensyICM20948, TeensyThreads, and AD9833 to seamlessly integrate sensor data with the Teensy controller. Operating at a CPU clock speed of 600 MHz and a baud rate of 115,200, the ET can execute a comprehensive suite of commands that enhance experimental flexibility. To facilitate the exploration of diverse use cases for the ET, we pre-defined several operational modes. For example, auto mode dynamically scales the maximum and minimum distances detected by the sensor to correspond with the motor’s full range of haptic feedback intensity. Manual mode enables experimenters to override sensor input, simulating haptic feedback at predefined distances, even in the absence of a physical object. Binary mode provides a simplified interaction paradigm, where haptic feedback is limited to a binary on/off response rather than a continuous gradient, allowing for investigations into sensory substitution under discrete stimulus conditions. A full description of these commands is provided in Table 2.

**Fig. 4:**
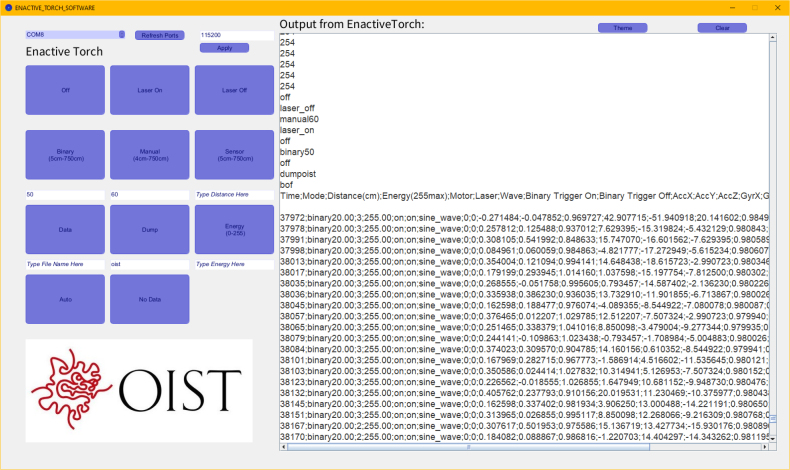
User interface for command delivery to the Enactive Torch. The interface features button controls and text-based input fields, providing an intuitive and accessible means of configuring and operating the device.

**Fig. 5:**
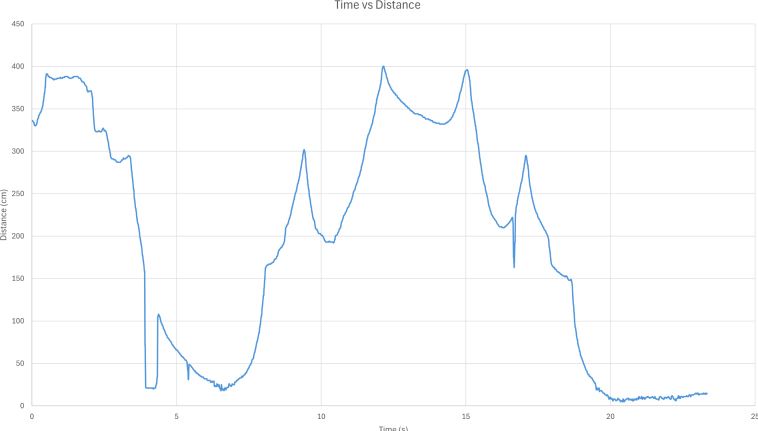
Distance–time relationship during object detection. The plot illustrates the variation in measured distance over time as the Enactive Torch is used to identify and track an object within its surrounding environment.

The ET user interface (Fig. 4) serves as the primary means of issuing commands and visualizing real-time performance metrics (Fig. 5). As the software is open-source, users can modify and extend the system by developing custom modes and triggers within the Arduino IDE to suit their experimental requirements. Moreover, the ET interface features user-accessible controls that allow seamless switching between operational modes, enhancing adaptability across experimental protocols. Additionally, saved datasets stored on the ET’s SD card can be reviewed (Table 3). In binary mode, the following parameters are recorded: timestamp (ms), the ET’s operational mode, distance measurements, PWM energy levels, motor vibration states, laser activity, waveform signals delivered to the motor, motor triggers, acceleration data, and roll, pitch, yaw, raw sensor readings, and device orientation. The various components within the ET operate at the following clock speeds: the accelerometer at 7 MHz, the waveform generator at 25 MHz, the SD card reader at 16 MHz, and the digital potentiometer at 10 MHz.

**Fig. 6:**
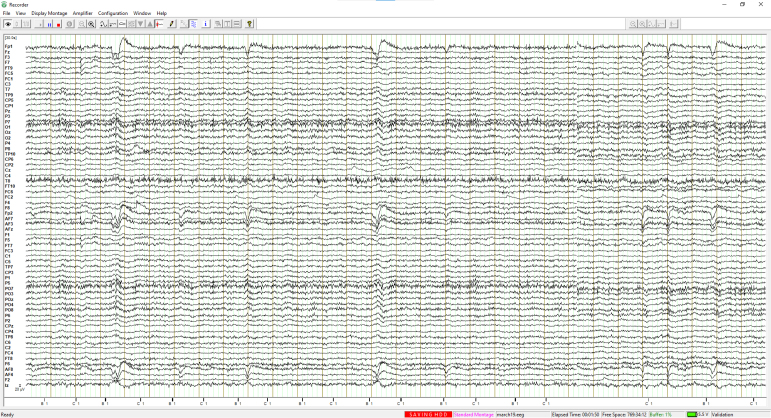
Triggering system of the Enactive Torch and its communication with an EEG setup. The plot illustrates how trigger events are transmitted between the Enactive Torch and the EEG recording system. Event flags displayed along the bottom denote key moments of interest during data acquisition.

A key feature of the ET is its integration with EEG systems via an external trigger interface. This was achieved using the Brain Vision TriggerBox (Prod.-Rev.02). The physiological measures, including EEG, electrocardiogram (ECG), and respiration, were recorded using Brain Vision Recorder Software (Version 1.20.0502; Brain Products GmbH, Gilching, Germany). This configuration allows the ET to deliver precisely timed triggers to the Brain Vision Recorder software, thereby marking critical events in an experimental protocol (Fig. 6). Triggers can be generated at the onset of trials, during the delivery of haptic feedback, when activating the laser, or at the initiation of specific commands — each providing a synchronized marker that enhances the interpretability of physiological data.

**Table 2:** Comprehensive command list for the Enactive Torch. Each entry specifies the command name, system response, trigger activation status, and a concise description of its role within the device’s operation.

Enactive Torch Commands			
Command	Response	Trigger	Description
Off	off	Yes	Turns off the ET
Laser On	laser_on	Yes	Turns on the laser pointer
Laser Off	laser_off	Yes	Turns off the laser pointer
Binary (5 cm–750 cm)	binary<dist>	Yes	Turns the ET to a binary off/max vibration at a certain distance in cm you choose
Manual (4 cm–750 cm)	manual<dist>	Yes	Continuously vibrates the motor at the distance in cm you indicated in the <dst>placeholder. (4cm≤##≤750cm)
Sensor (5 cm–750 cm)	sensor<dist>	Yes	Directly scales the max & minimum vibration intensity to the max distance (cm) of your choice & the minimum sensor distance. (5cm≤##≤750cm)
Data	data<file_name>	Yes	Write all the incoming data on to the SD Card (only binary)
Dump	bof <file content line 1><file content line 2>... <file content line n>eof	No	Stream the content of the file to serial port and turn the ET off
Energy (0 – 255)	energy<number>	No	Change the power of the motor (0 is no power, 255 is max power and default)
Auto	auto	Yes	Directly scales the max & minimum vibration intensity to the max & minimum sensor distance. (4cm≤##≤750cm)
No Data	nodata<file_name>	Yes	Interrupt writing the data to SD Card

**Table 3:** The sample dataset includes recorded parameters such as time, operational mode, measured distance, power output, motor status, laser and trigger states, and accelerometer readings displayed in real time.

## 3 Design files summary

The design files required to reproduce the Enactive Torch are summarized in Table 4 and are available in the project repository.

**Table 4:** Design files released with the project.

Design file name	File type	Open source license	Location of the file
ET 5.7 Bottom	CAD	CC BY	https://osf.io/vrsya/overview
ET 5.7 Laser Holder	CAD	CC BY	https://osf.io/vrsya/overview
ET 5.7 SD Holder	CAD	CC BY	https://osf.io/vrsya/overview
ET 5.7 Top	CAD	CC BY	https://osf.io/vrsya/overview
ET 5.7 Window	CAD	CC BY	https://osf.io/vrsya/overview
External Vibration Case 4.0	CAD	CC BY	https://osf.io/vrsya/overview
Motor Holder 2.0	CAD	CC BY	https://osf.io/vrsya/overview
ET 5.7 Bottom Laser Holder Bottom	.STL	CC BY	https://osf.io/vrsya/overview
ET 5.7 Bottom Laser Holder Top	.STL	CC BY	https://osf.io/vrsya/overview
ET 5.7 Bottom SD Card Holder	.STL	CC BY	https://osf.io/vrsya/overview
ET 5.7 Bottom	.STL	CC BY	https://osf.io/vrsya/overview
ET 5.7 Top Window	.STL	CC BY	https://osf.io/vrsya/overview
ET 5.8 Top	.STL	CC BY	https://osf.io/vrsya/overview
External Vibration Case 4.0 bottom	.STL	CC BY	https://osf.io/vrsya/overview
External Vibration Case 4.0 top	.STL	CC BY	https://osf.io/vrsya/overview
Motor Holder 2.0	.STL	CC BY	https://osf.io/vrsya/overview

## 4 Bill of materials summary

The complete bill of material is included as “ET BOM.pdf” in the “Instructions” folder of the repository (https://osf.io/vrsya/overview). The PDF lists the cost, location of purchase, and other additional information pertaining to the equipment. The ET printed circuit board can be manufactured through JLCPCB.com or through other corporations, and the Gerber, BOM, and CPL files are required. These files are found in the “Circuit Board Manufacturing Files” folder.

## 5 Build instructions

### 5.1 Material

The materials required to construct the ET were sourced from a diverse range of suppliers, including Sparkfun, Marutsu, Monotaro, Mouser, Digikey, Misumi, AliExpress, JLCPCB, and Amazon. During development, all components were shipped to Japan, with delivery times ranging from one to four weeks. At the time of assembly, each component was also readily available from multiple international suppliers, ensuring that the sourcing process should not be geographically restrictive for replication in other regions. No shortages, tariff-related delays, or other supply chain constraints were encountered during procurement. The complete bill of materials (BOM), including detailed part specifications and sourcing links, is provided in the project repository as referenced in Section 4.

### 5.2 Hardware assembly

As noted in Section 2.1, the hardware casing of the ET is composed of custom 3D-printed parts designed to provide structural stability and component integration. The majority of these components were fabricated in 3201PA-F Nylon using SLS, while the transparent access window (ET 5.7 Window) was produced in 8001 Resin via SLA to permit visual inspection. For proper assembly, the following parts were printed in 3201PA-F Nylon: ET 5.7 Bottom Laser Holder Bottom, ET 5.7 Bottom Laser Holder Top, ET 5.7 Bottom SD Card Holder Top, ET 5.7 Bottom, ET 5.7 Top Window, ET 5.8 Top, and Motor Holder 2.0. In addition to the 3D-printed parts, threaded screw inserts (M3 × 2 mm, M3 × 7 mm, and M2 × 3 mm) are required to secure components and ensure long-term mechanical reliability.

**Fig. 7:**
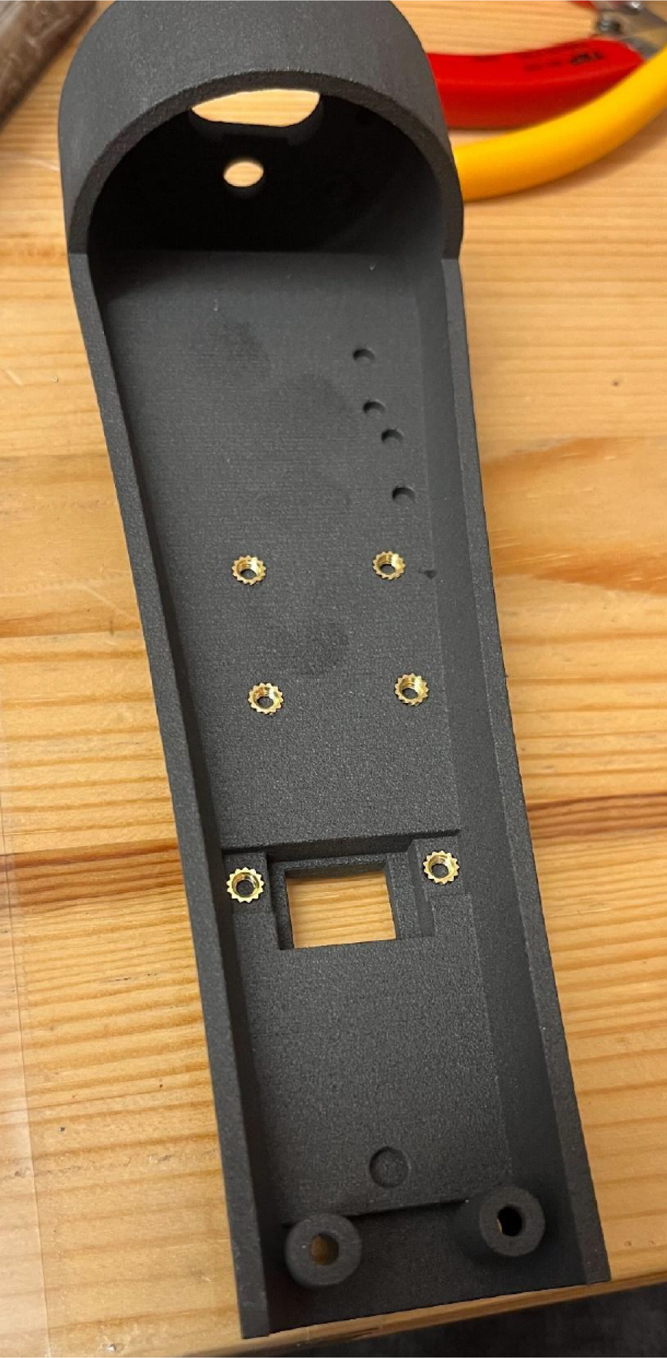
Screw inserts embedded within the 3D-printed body of the Enactive Torch. Metal threaded inserts are heat-set into the printed enclosure to provide durable fastening points for repeated assembly and disassembly.

1. Screw Insert Installation: The accompanying blueprints (“ET 5.7 Blueprints.PDF”) specify the precise locations for all screw inserts (https://osf.io/vrsya/overview). To install each insert, position it over the designated hole and carefully press it into place using a heated soldering iron. Care must be taken to avoid overheating or damaging the surrounding 3D-printed material. On page 1 of the blueprints (“Bottom”), seven M3 × 3 mm inserts and eight M2 × 3 mm inserts are required. The seven M3 × 3 mm inserts are placed in four holes forming a square pattern, two holes at the SD Holder location, and one hole just beyond the SD Holder (Fig. 7). The eight M2 × 3 mm inserts are placed in the four smaller holes below the square pattern and four additional holes in the LiDAR and laser pointer mounting areas (“Section B–B” in the blueprints). On page 2 (“Top”), six M3 × 3 mm inserts and two M3 × 7 mm inserts are required. The deeper holes indicated in the blueprints should receive the M3 × 7 mm inserts. The M3 × 3 mm inserts are distributed as follows: two in the motor area, one in the window area, and three above the motor and window regions as shown in the drawings.
2. Window Installation: The transparent window is secured using a single M3 × 5 mm screw. Position the window within the designated recess of the ET 5.8 Top component, ensuring a precise and flush fit. Confirm that the curvature of the window aligns seamlessly with the external contour of the 3D-printed shell to maintain both structural integrity and visual continuity.

### 5.3 Electronics assembly

As described in Section 4, the ET printed circuit board (PCB) was fabricated via JLCPCB.com, a commercial PCB manufacturing service. Comprehensive, step-by-step instructions for file submission, panelization options, and production settings are available in the project repository to ensure reproducibility.

For PCB assembly, the following components are required: male header pins, Teensy 4.0 microcontroller, accelerometer module, two Adafruit Mono 2.5 W Class D audio amplifiers, two AD9833 DDS wave signal generator modules, PCB LiDAR plug (through-hole mount), two surface-mount digital potentiometers, through-hole transistors, Micro USB plug (V8UP-4C), two LiPo charger/booster modules (5 V/1 A), Teensy microSD adapter, TFMini-S micro LiDAR module, laser pointer, three 1800 Ω through-hole resistors, insulated hookup wire, a soldering iron, and appropriate solder. Depending on manufacturing availability, certain components (e.g., male header pins, transistors) may arrive pre-installed by the supplier.

**Fig. 8:**
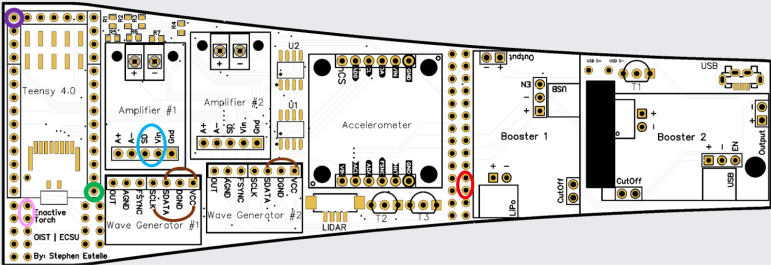
Color-coded highlights indicate key signal connections: the purple circle represent the Teensy SCLK line, the green circle denote Teensy ground (G) connections, the red circle marks unused pins, the blue circle shows wired interconnections, brown arcs correspond to 1800Ω resistor links, and a pink circle indicates the D+ and D– USB data lines.

**Fig. 9:**
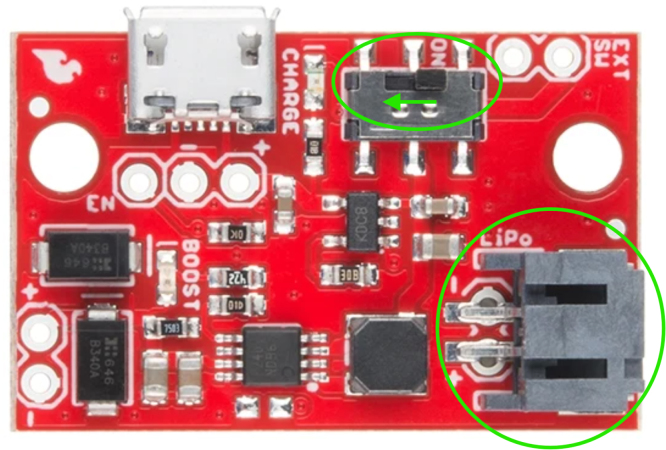
LiPo charger/booster module showing the power switch and JST battery connector. Green circles highlight the locations of the LiPo power switch and the corresponding JST port used for battery connection.

**Fig. 10:**
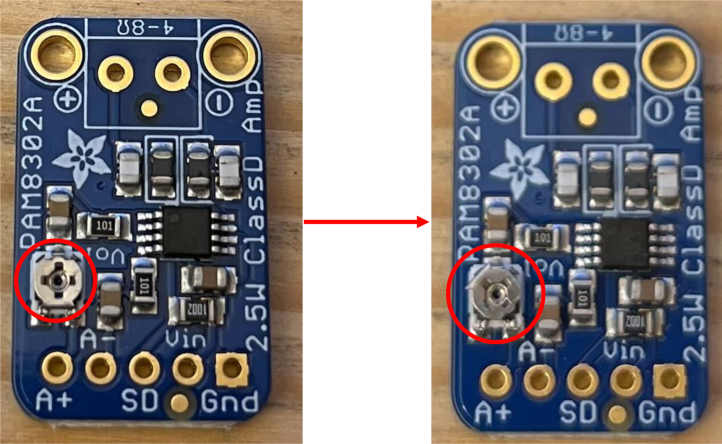
Adjusting the amplifier to its maximum output level. This step illustrates the configuration of the amplifier to achieve peak signal amplification for optimal haptic feedback intensity.

**Fig. 11:**
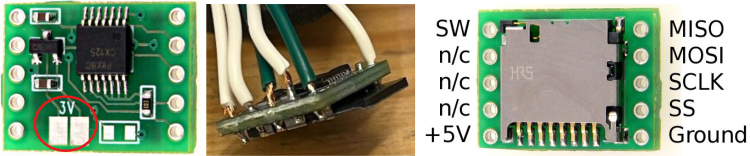
3V pads and corresponding wire attachments used to interface the adapter with the Enactive Torch circuit.

**Fig. 12:**
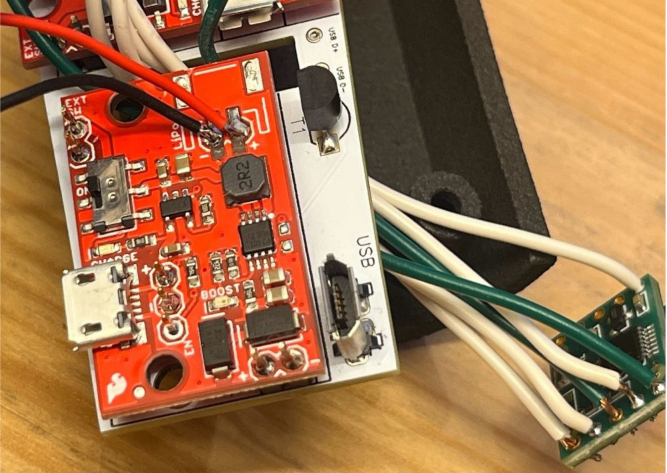
The SD adapter leads are passed beneath the board opening to maintain secure placement and reduce strain on soldered connections.

**Fig. 13:**
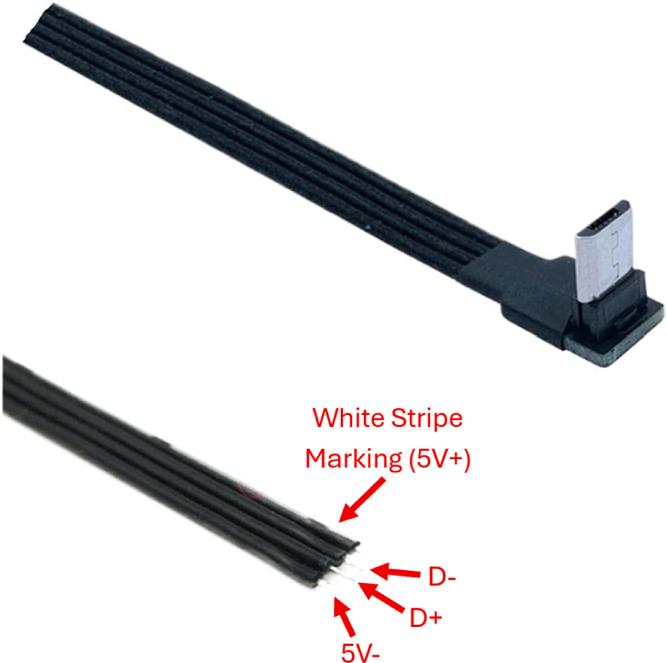
The data lines (D– and D+) used for USB communication between the Enactive Torch and external devices through the micro USB Plug.

**Fig. 14:**
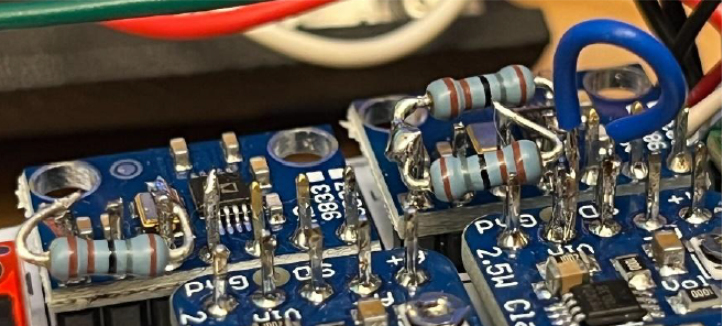
Resistors and connecting wires are arranged on the circuit board to complete signal and power pathways within the Enactive Torch system.

**Fig. 15:**
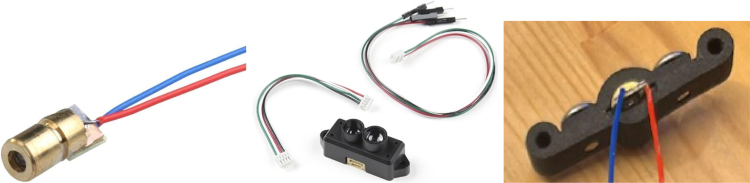
Laser pointer and LiDAR wiring.

**Fig. 16:**
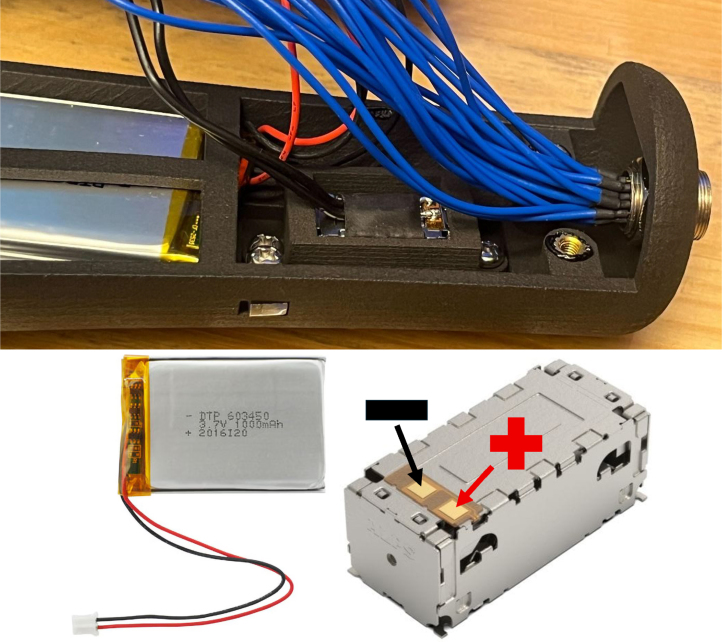
The internal arrangement of the LiPo battery, motor, and 20-pin connector, highlighting their placement and wiring within the device’s top enclosure.

**Fig. 17:**
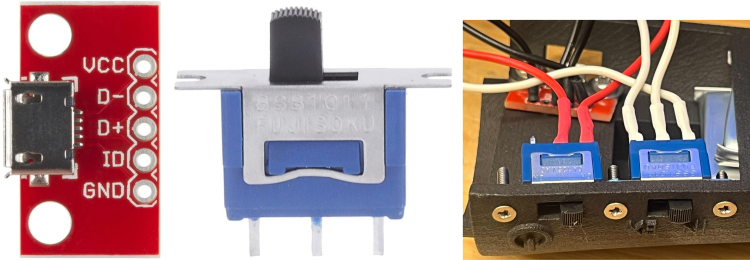
The connection layout between the MicroB USB breakout board, power switch module, and associated wiring used for system power control and data communication.

**Fig. 18:**
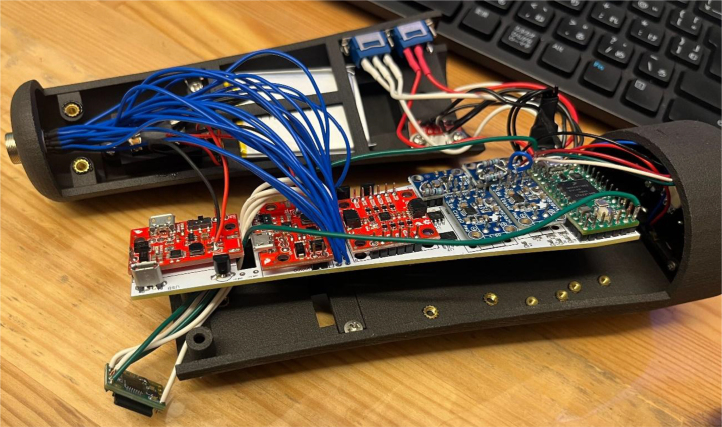
All major electronic modules interconnected within the Enactive Torch, illustrating the complete internal wiring and component integration.

1. Install the digital potentiometers: Solder the two surface-mount digital potentiometers at positions U1 and U2 (Fig. 8). Align the orientation mark (small corner dot) on each device with the corresponding mark on the PCB silkscreen. The use of solder paste is recommended for accuracy and thermal control.
2. Install male header pins: Fit and solder header pins as follows — 12 pins for the accelerometer, 7 pins for each wave generator, 7 pins for each amplifier, 9 pins for each LiPo charger/booster, and 33 pins for the Teensy 4.0 — for a total of 91 pins (Fig. 8).
3. Mount and solder modules: Attach the accelerometer first, ensuring pin labels match between the top of the module and PCB, then solder in place. Repeat for the wave generators and Teensy 4.0.
4. Prepare LiPo charger/boosters: Before installation, remove the LiPo battery JST port connectors using cutters or pliers (Fig. 9). Detailed guidance is available from the manufacturer at sparkfun.com. Set the adjacent USB-side switch to the “off” position for now (Fig. 9) Afterwards, place on to male header pins and solder the connection.
5. Prepare and install amplifiers: Prior to soldering, rotate the gain adjustment knob fully clockwise to enable maximum output (Fig. 10). Install each amplifier in its designated footprint labeled “Amplifier #1” and “Amplifier #2” on the PCB (Fig. 8).
6. Preparing SD Card adapter: The bottom side of the Teensy microSD adapter requires the 3 V solder pads to be connected via soldering to bridge the pads together. Additionally, the microSD adapter requires six insulated wires, each 15 cm in length, to be soldered to specific pins. Following the pin diagram on the manufacturer’s website and Fig. 11, connect the wires to the following terminals: +5V, Ground, SS, SCLK, MOSI, and MISO. Insert the wires into the adapter’s solder holes from the bottom side opposite the metal SD card holder to ensure clearance and prevent mechanical interference. After soldering, route the wires from the underside of the PCB through the rectangular cutout between the LiPo charger/booster modules (Fig. 12). Solder the wire ends to the designated location on the PCB, as indicated in Table 5 and Fig. 8. This routing minimizes strain on the solder joints and maintains accessibility for subsequent assembly steps.
7. Preparing Micro USB Plug (V8UP-4C): The Micro USB connector will interface with the Teensy’s onboard USB port; however, prior to insertion, the two center data lines (D+ and D-) must be isolated. The D- line is identified as the central conductor adjacent to the wire with white stripe marking. The D+ line is adjacent to the D- line (Fig. 13). On the underside of the ET PCB, near the Teensy’s USB port, locate the two plated through-holes labeled “D-” and “D+” (Fig. 8). Solder the corresponding USB conductors to these holes from the top of the ET PCB, ensuring correct polarity and secure mechanical contact. Once soldered, the Micro USB plug may be inserted into the Teensy’s USB port.
8. Supplemental resistors and wire: To enhance signal stability within certain modules, specific resistive and wiring modifications are required. For Wave Generator #1, solder two 1800 Ω through-hole resistors — one bridging the VCC and SDATA pins, and another bridging the VCC and SCLK pins. For Wave Generator #2, install a single 1800Ω through-hole resistor between the VCC and SDATA pins (Figs. 8 and 14). Additionally, connect a short length of insulated wire between the SD and Vin pins on Amplifier #1. These modifications should be performed with precision to ensure reliable electrical connections and to minimize mechanical stress on the components.
9. LiDAR and Laser Pointer: The LiDAR module and laser pointer require straightforward wire termination and soldering to the designated PCB pin locations. The laser pointer has two leads—red (positive) and blue (negative)—which should be routed and soldered accordingly (Fig. 15). For the LiDAR, use the supplied four-conductor cable fitted with individual male pin headers. Insert the white connector into the LiDAR socket, then cut off the black male tips on the opposite end and strip approximately 2–3 mm of insulation to expose the copper conductors using wire strippers. The LiDAR cable comprises four colored wires: red (VCC), black (GND), green (TX), and white (RX). Table 5 identifies the corresponding ET PCB pin locations for each connection. As with the Micro USB connector, pin labels are printed on the underside of the PCB near the Teensy microcontroller. Insert each wire through the top side of the board and solder on to the underside, consistent with the procedure used for the other wired connections. To mount, begin by tapping the holes on the ET 5.7 Bottom Laser Holder Bottom with an M3 tap. Position the laser pointer within the central recess of the holder, ensuring it rests securely in the designated divot. The laser’s solder board with the attached wires should lie parallel to, and just above, the small wall located at the rear of the divot. Place the ET 5.7 Bottom Laser Holder Top over the assembly and secure both holder components around the laser pointer using an M3 × 5 mm screw. Once the laser pointer is clamped, use M2 × 12 mm screws to fasten the complete holder assembly, along with the LiDAR module, onto the screw inserts of the ET 5.7 Bottom component. When inserting, orient the LiDAR module upside down so that its wiring does not interfere with the laser pointer.
10. LiPo Battery: These components require particular care during installation to ensure correct placement and wiring (Fig. 16). Improper assembly may result in device malfunction or failure. For the LiPo batteries, begin by cutting off the white plug at the end of each power lead and carefully stripping the insulation to expose the conductive cores. Slide each battery into the designated cage-like holder on the upper half of the ET (ET 5.8 Top), ensuring the wires are routed through the back of the cage and extend beyond it. It is critical to maintain the pairing of each red (positive) and black (negative) lead—do not mix wires between batteries. Following Fig. 9 and Fig. 12, solder each red wire to the LiPo+ pad and each black wire to the LiPo- pad. Each battery should be wired to a separate LiPo Charger/Booster module to distribute the load and ensure stable operation.
11. Motor Installation: The motor must be installed with particular care due to the fragility of its soldering pads. As shown in Fig. 16, the pad located nearest to the motor housing entrance corresponds to the positive terminal, while the opposite pad serves as the negative terminal. Solder 12 cm insulated wires to these pads, ensuring the wires are laid flat against the motor casing to reduce strain. Excessive force or tension should be avoided, as this may detach the pads from the motor body. Once soldered, cut a 1 cm length of appropriately sized heat-shrink tubing and apply it around the solder joints, securing both the motor terminals and the wires for mechanical reinforcement. Next, apply a section of double-sided adhesive tape to the underside of the motor and insert the assembly into the 3D-printed “Motor Holder 2.0”, routing the wires through the top opening. Secure the motor holder to the designated screw inserts in the chassis, as shown in Fig. 16. Finally, solder the motor’s positive lead to pin #27 and the negative lead to pin #28 on the PCB, as listed in Table 5.
12. Panel Mount Installation: The 20-pin male panel mount is inserted through the rear access port of the ET’s upper housing. Ensure that the hex nut and lock washer are positioned inside the ET casing prior to tightening. Using pliers, firmly secure the hex nut to prevent loosening during vibration or operation. Trim the attached blue wires to approximately 15 cm (or shorter, as needed), and carefully note the identification number printed attached to each wire. These numbers directly correspond to the matching pin labels on the PCB. For example, wire labeled “1” must be soldered to pin “1” on the board. Continue this process sequentially until all wires are attached. As each connection is completed, remove the temporary white number labels to avoid clutter and confusion during final assembly.
13. MicroB USB Breakout and Switches: The MicroB USB breakout (Fig. 17) provides both recharging capability and access for firmware updates. Four insulated wires (13 cm each) must be soldered to the breakout at the VCC, D-, D+, and GND terminals - skip the ID pin. These wires should then be routed to their corresponding pins on the PCB as indicated in Table 5. Two switches are also required to enable system control. The internal/external motor selector (Switch #1) requires three individual 13 cm wires to be soldered to each of its poles. The power switch (Switch #2) requires two 13 cm wires — one connected to the center pole and the other to either of the two remaining poles. All switch conductors should be routed and soldered to the appropriate pads on the PCB as specified in Table 5. Once all wiring is complete, secure the USB breakout and the two switches to the chassis. Use two M3 × 5 screws with washers for the USB breakout, and three M2 × 12 screws with matching M2 nuts for the switches. Tighten gently but firmly to ensure reliable mounting without overstressing the plastic housings.
14. SD Card Reader Holder: Mount the SD card reader into the lower half of the ET (ET 5.7 Bottom). Begin by positioning the SD card reader in the designated slot indicated in the blueprints (page 1). Ensure that no SD card is inserted during installation, as this will prevent proper placement. The square metallic housing of the reader should face downward toward the external shell, with the card insertion opening oriented toward the embossed text “PULL” on the ET bottom shell. Once aligned, secure the SD card reader by fastening the ET 5.7 Bottom SD Card Holder over its backside into the screw inserts provided with M3 x 5 mm screws. To improve stability, a thin piece of polyethylene (PE) or anti-static foam may be placed between the holder and the SD card reader to ensure the component is firmly pinned in position.
15. PCB Installation: To secure the PCB onto the bottom ET shell, use five M3 × 10 mm screws and four M2 × 8 mm screws. Align the four mounting holes of the accelerometer with the corresponding inserts arranged in a square pattern, the four mounting holes of the two amplifiers with their designated positions, and the single hole located beneath the rearmost LiPo Charger/Booster module (Figs. 7 and 18). Once properly positioned, the PCB should cover the SD card reader and its holder. Fasten the amplifiers using the four M2 × 8 mm screws, and secure the accelerometer and LiPo Charger/Booster with the five M3 × 10 mm screws. This sequence ensures proper alignment, mechanical stability, and consistent electrical interfacing.
16. Final Enclosure Assembly: To complete the ET assembly, position the top half of the enclosure by engaging its front lip beneath the tunnel of the bottom half. Carefully route all wiring inside to prevent pinching or strain before closure. Finally, use two M3 × 10 mm screws through the designated holes on the outer shell of the bottom half to lock both halves together.

**Table 5:** Location and labeling of each module connection pin used to interface with the Enactive Torch.

Module adapter pin	Soldering location
SD Adapter (MOSI)	21
SD Adapter (MISO)	22
SD Adapter (+5V)	23
SD Adapter (SS)	24
SD Adapter (Ground)	Directly to Teensy Pin G
SD Adapter (SCLK)	Directly to Teensy Pin 13
Laser Pointer (Red)	LZR+
Laser Pointer (Blue)	LZR-
LiDAR (Red)	TFM+
LiDAR (Black)	TFM-
LiDAR (Green)	Tx
LiDAR (White)	Rx
Motor +	27
Motor -	28
MicroB USB (VCC)	VCC
MicroB USB (D-)	D-
MicroB USB (D+)	D+
MicroB USB (GND)	GND2
Switch #1 (EX)	EX
Switch #1 (Center)	OUT-
Switch #1 (IN)	IN
Switch #2 (Center)	GND
Switch #2 (Outer)	EN

### 5.4 External hardware assembly

Assembly of the external components for the ET requires the following parts: USB serial connector, AUX wire, four BNC trigger plugs, general-purpose wire, AUX plug, five M2 × 3 mm screw inserts, two M3 x 2 mm screw inserts, 20-pin connector cable, lever-style wire connectors, splicing wire connectors, double-sided Velcro, Spiral Cable Wrap, five M2 × 12 mm screws, two M3 x 8 mm screws, motor, Motor Holder 2.0, External Vibration Case 4.0 Bottom, and External Vibration Case 4.0 Top. For the 25-pin connector, only cables numbered 1 and 13–20 are utilized (Table 6), yielding nine active conductors that must be terminated to their corresponding components. All remaining conductors on the 25-pin connector may be disregarded or trimmed to prevent interference.

**Fig. 19:**
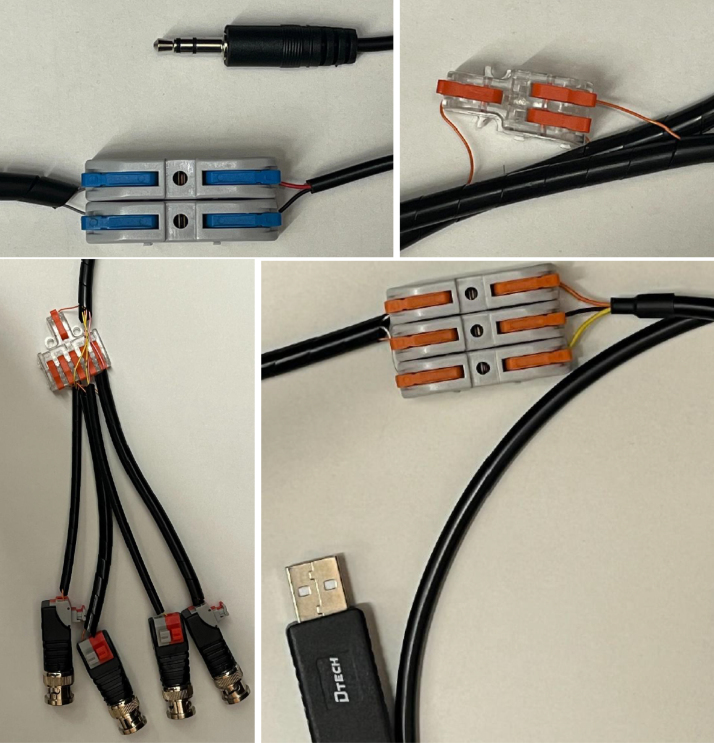
Wire #13 and #14 are connected to the black and red AUX wire, respectively (Top Left). Ground is divided to two (Top Right). BNC plugs each receive a ground connection, and a signal pin (Bottom Left). Three pins required to connect USB Serial Connector (Bottom Right).

1. On the 20-pin connector cable, identify the designated wires using the white numerical tags. Separate and remove wires numbered 2 through 12, as these conductors are not required for assembly. Trim them cleanly and discard to reduce clutter and eliminate the risk of electrical interference during operation.
2. On the AUX cable, remove the white wire, as it is not required for assembly, and strip the black and red wires for more conductive exposure. On the 20-pin connector cable, wires numbered 13 and 14 must each be connected to separate lever wire connectors (Fig. 19). Strip the ends of these wires to expose the conductive cores and insert them firmly into the lever connectors. Opposite wire #13, insert the black conductor from the AUX cable; opposite wire #14, insert the red conductor. Ensure all terminations are fully seated within the connectors to maintain reliable electrical contact and reduce the risk of disconnection during operation.
3. Wire #1 must be divided into two outputs using a 1-in-2-out splicing connector (Fig. 19). Begin by cutting Wire #1 to half its original length. Prepare a length of general-purpose wire equal to the freed section of Wire #1. Strip the insulation from the cut ends of Wire #1 and from both ends of the general-purpose wire to expose the conductive cores. Insert the section of Wire #1 still attached to the 20-pin connector into the single-entry side of the splicing connector. On the dual-entry side, insert the freed end of Wire #1 and the general-purpose wire in to separate entry points. Ensure all conductors are fully seated within the connector to establish secure electrical contact and prevent accidental disconnection during operation.
4. Prepare the BNC trigger plugs by connecting wires #17, #18, #19, and #20 to the positive terminals of four separate BNC plugs (Fig. 19). From the previous wiring step, take one of the available ground conductors and insert it into the single-entry side of a 1-in-5-out splicing connector. Cut and strip four short lengths of general-purpose wire and insert each into one of the multi-entry ports on the opposite side of the splicing connector. The fifth entry port should remain unused. Connect the free ends of these four wires to the negative terminals of the corresponding BNC plugs, ensuring each plug receives one positive and one negative connection. Verify all conductors are fully seated to guarantee reliable electrical contact and minimize the risk of disconnection during operation.
5. The USB serial connector requires only three conductors for operation. Remove the black female pin headers from all wires, then cut and discard all colored leads except for the orange (TXD), black (ground), and yellow (RXD) wires. Strip the insulation from these three conductors and insert each in a lever wire connector. Connect wire #15 to the orange TXD lead, wire #16 to the yellow RXD lead, and the additional ground conductor from the previous step to the black ground lead (Fig. 19). Ensure each connection is firmly seated within the lever connectors to provide secure electrical contact and prevent accidental disconnection during use.
6. To ensure proper protection and organization, group related wires together and secure them using spiral cable wrap. This bundling method reduces the risk of tangling, minimizes mechanical strain on individual conductors, and prevents accidental pulling during assembly or operation.
7. The final stage is the assembly of the external vibration case. Begin by inserting five M2 × 3 mm screw inserts into the designated holes on the External Vibration Case 4.0 Top. Use a soldering iron to carefully heat and press the inserts into place, ensuring they seat flush without damaging the surrounding material. Repeat this process for two M3 × 2 mm screw inserts on the External Vibration Case 4.0 Bottom. Following the same procedure described for the internal ET motor in Step 11 and Fig. 16, solder approximately 5 cm of insulated wire to the positive and negative pads on the motor, laying the conductors flat against the housing to reduce strain. Prepare the leads with exposed ends for subsequent soldering, then secure them to the motor with 1 cm of heat-shrink tubing to reinforce the joints. Affix double-sided tape to the motor base and mount the motor onto the External Vibration Case 4.0 Bottom using two M3 × 8 mm screws and the Motor Holder 2.0, routing the wires through the opening in the top of the holder. Finally, solder the negative motor lead to the first pin of the AUX plug (closest to the AUX opening) and the positive lead to the adjacent second pin (second closest to the AUX opening). Cover each solder joint with heat-shrink tubing to prevent accidental shorting. Insert the AUX plug through the designated hole in the case top, secure it in place with the screw-on ring, and neatly arrange the wires within the enclosure. Close the assembly by fastening the two halves of the case together with M2 × 12 mm screws through the bottom-half mounting holes.

**Table 6:** Pin Connector Cable wires and its corresponding components.

Wire number	Component
1	Ground
13	External Motor (Negative)
14	External Motor (Positive)
15	TX
16	RX
17	BNC (Positive)
18	BNC (Positive)
19	BNC (Positive)
20	BNC (Positive)

## 6 Operation instructions

The ET can be operated in multiple modes using a graphical user interface (GUI) and command line controls via serial communication. Below are the standard operating procedures:

**Fig. 20:**
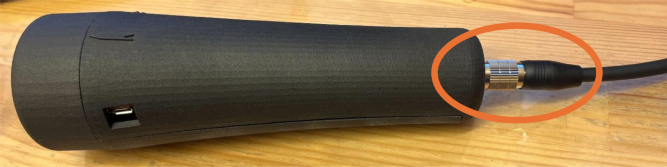
Enactive Torch interfaced with the wire connector, showing the alignment and secure attachment of the connecting terminals.

**Fig. 21:**
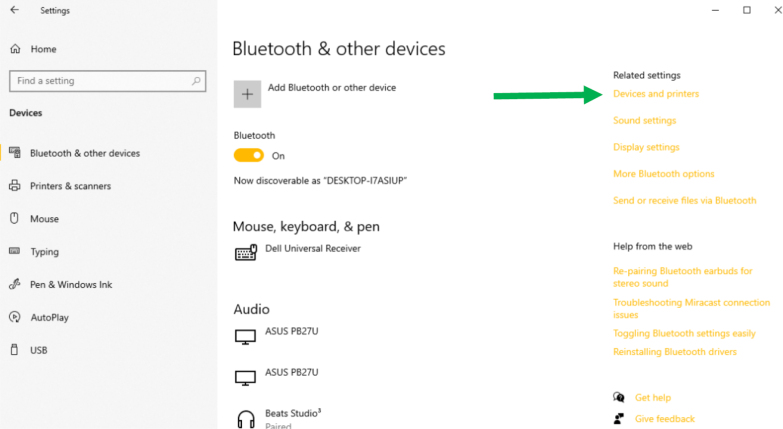
“Devices and printers” is located under the “Related settings” column.

**Fig. 22:**
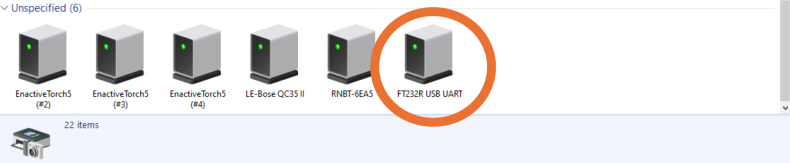
The Enactive Torch USB port number is located in the UART device.

**Fig. 23:**
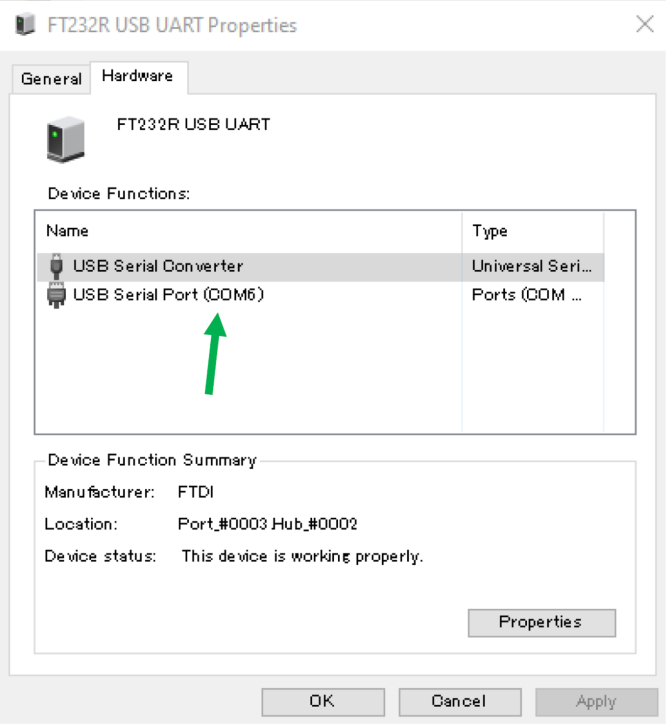
COM number location.

### 6.1 Powering on the device

• Ensure the LiPo batteries are fully charged. When plugged in, a blue light emits when charging, and disappears when fully charged.
• Toggle the main power switch to activate the Teensy microcontroller and peripherals.
• Observe the LED through the transparent side panel to verify device status. If you see a red light, the ET is powered on.

### 6.2 Setting up ET

• You first need to connect the ET to its respective ET Wire connector. Ensure that you do not over tighten, and just tight enough until the plug stops spinning. Plug the opposite end of the ET Wire connector USB in to a computer (Fig. 20).
• To use the ET in Arduino IDE, the port number that the ET Wire connector is using needs to be determined. For this example, a Window’s computer is utilized. On the Windows computer, go to your “Bluetooth and other devices” settings, and click on “Devices and printers” (Fig. 21).
• When you plug in the USB to the ET Wire, “FT232R USB UART” should pop up. If this is the first time plugging in the USB to the computer, it may take a couple seconds to connect. Right click the icon, and go in to the “properties” (Fig. 22).
• In “properties”, click on the tab “Hardware”, and there should be a small list with something that says “USB Serial Port (COM#)”. That COM number is needed to use the ET. After noting the COM number, close the properties menu, the devices and printers menu, and the bluetooth menu (Fig. 23).

### 6.3 Connecting to Arduino IDE

**Fig. 24:**
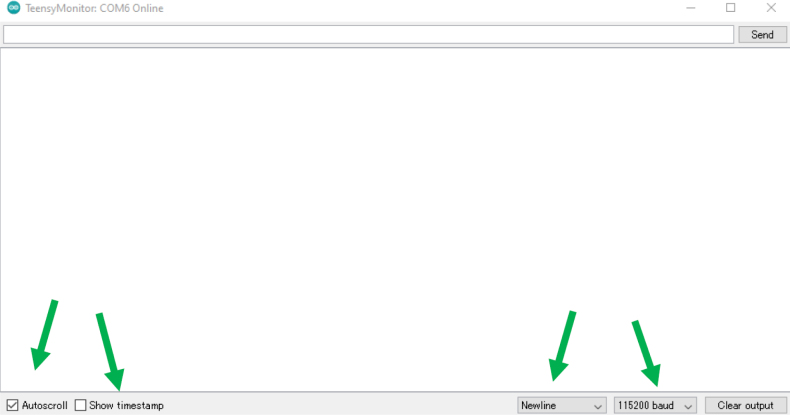
Settings used during Enactive Torch operation.

**Fig. 25:**
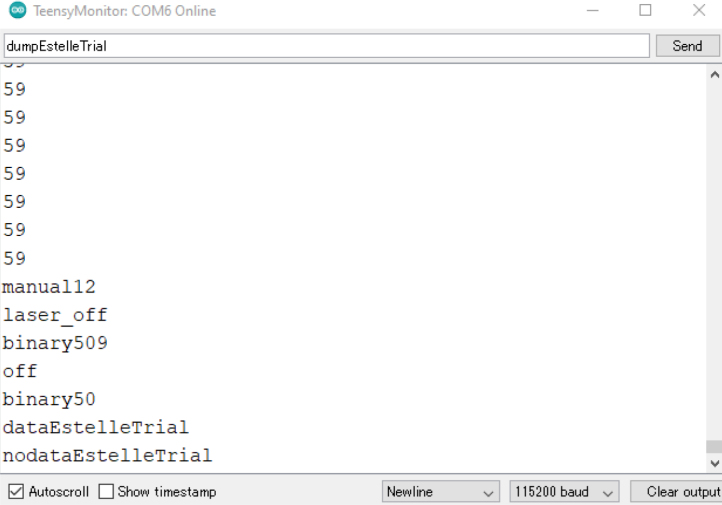
Measured distances alongside the active modes of the Enactive Torch during data collection.

• In the Arduino IDE software, under “Tools ⇒ Ports”, select the COM that represents the ET USB. This is the COM that is needed to use whenever you want to send commands to the ET. This COM number is different between different computers, and different USB wires. If different ETs are used on the same ET External Wire, the COM will stay the same between the ETs.
• With the correct COM selected, select “Tools ⇒ Serial Monitor” and have the settings as shown in Fig. 24.
• Turn on the ET to begin seeing the real-time distance perceived from the sensor (Fig. 25).

### 6.4 Selecting operation mode

**Fig. 26:**
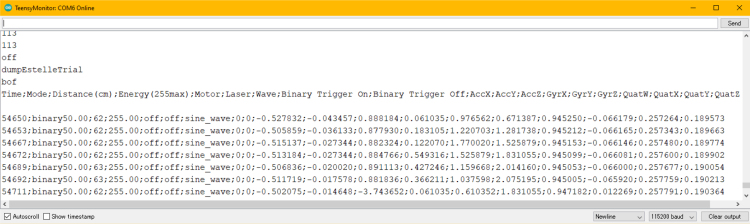
Captured parameters, including time, signal state, sensor readings, and corresponding binary outputs generated during device activity.

**Fig. 27:**
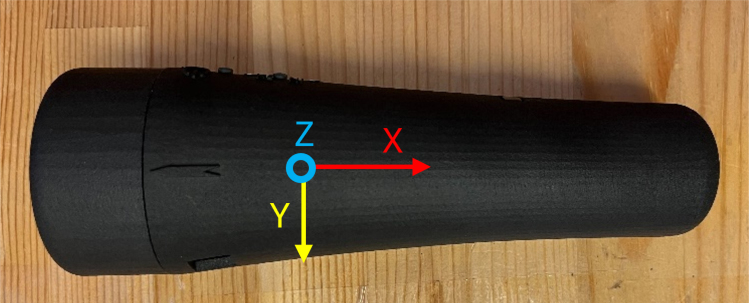
Illustration of how spatial data is aligned and interpreted with respect to the Enactive Torch’s orientation during operation.

• The ET starts in auto mode. Type in the commands in the bar and click “send” to send commands. The commands and their description is listed in Table 2. Available modes include:
  – auto
  – manual##
  – binary##
  – sensor##
  – binary##
  – off
  – laser_on
  – laser_off
  – energy##
  – data < file_name >
  – nodata
• The data collection is only possible in binary mode as of now (Figs. 26 &27). You must have a micro SD card in the device to collect data. The data you receive are:
  – Time - The time in milliseconds since the device has been on
  – Mode - The current mode the device is running in
  – Distance (cm) - The perceived distance at that moment
  – Energy (255max) - The current active energy level
  – Motor - If the motor is off or on
  – Laser - If the laser is off or on
  – Wave - The wave being generated (sine is default)
  – Binary Trigger On - When the motor starts vibrating, a trigger (1) is sent to indicate the timestamp
  – Binary Trigger Off - When the motor stops vibrating, a trigger (1) is sent to indicate the timestamp
  – AccX - Acceleration in X direction (Forward and back of the arrow)
  – AccY - Acceleration in Y direction (Moving left and right of the arrow)
  – AccZ - Acceleration in Z direction (Moving up and down of the arrow)
  – GyrX - Rotation about X axis
  – GyrY - Rotation about Y axis
  – GyrZ - Rotation about Z axis
  – QuatW - Quaternion Values for rotation
  – QuatX - Quaternion Values for rotation
  – QuatY - Quaternion Values for rotation
  – QuatZ - Quaternion Values for rotation

### 6.5 External motor and triggers

**Fig. 28:**
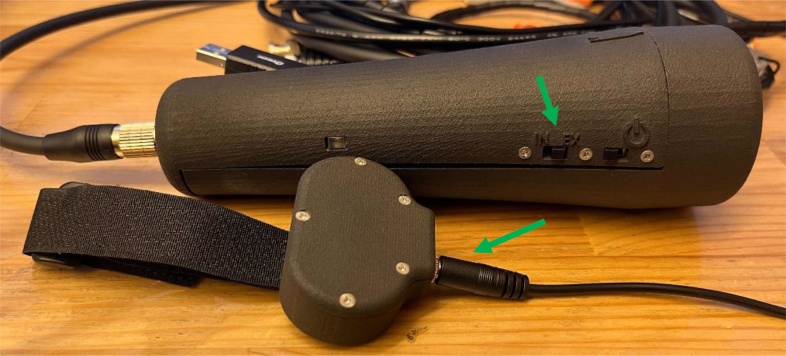
Switching between the internal and external motors.

**Fig. 29:**
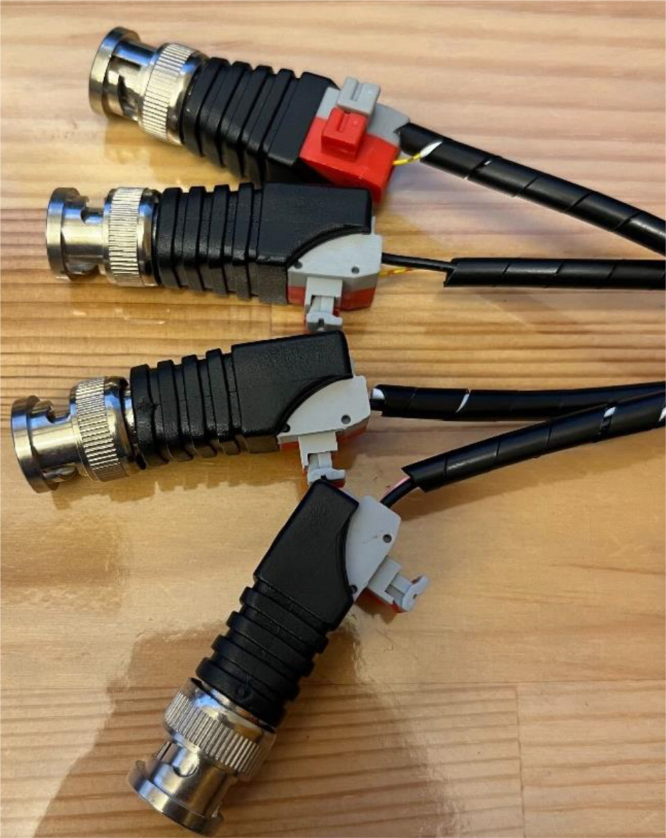
Connector BNC plugs used to deliver trigger data to EEG trigger box.

• On the ET Wire connector, plug in the external Motor and toggle the ET switch to change between External and Internal Motors (Fig. 28).
• On the External ET Wire, connect the ET trigger pins into your EEG trigger box to receive triggers (Fig. 29).
• Confirm that trigger pulses are synchronized with event timing (e.g., trial onset or motor activation).

### 6.6 Data logging

• Collected data (timestamp, mode, distance, PWM values, accelerometer readings, etc.) are logged to the onboard SD card.
• Retrieve and analyze this data using the GUI or post-processing software.

### 6.7 Powering down

• Exit the Arduino IDE and disconnect the USB cable.
• Switch off the device to preserve battery life.
• Avoid dropping the device or hitting nearby objects.

### 6.8 Safety considerations

• Avoid prolonged exposure of the skin to vibrating surfaces.
• Never short-circuit the battery terminals.
• Ensure motors are securely mounted to prevent vibration-related dislodgement.

## 7 Validation and characterization

**Fig. 30:**
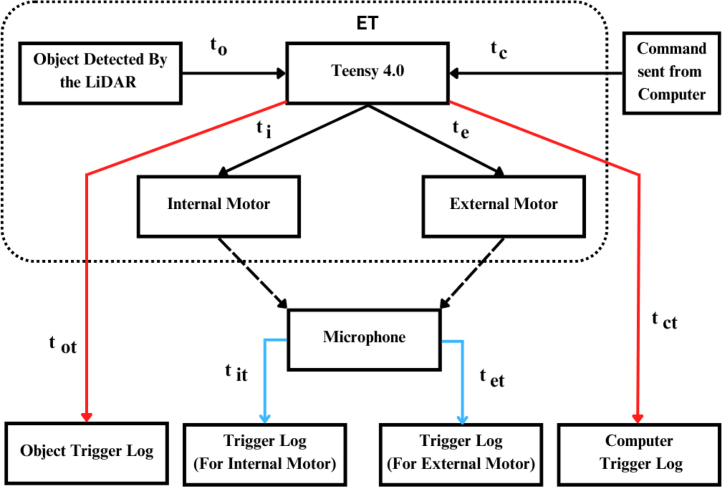
Demonstration of the integration between the Enactive Torch and the EEG acquisition system, highlighting the timing alignment used to verify accurate event synchronization.

This section presents a validation of the ET, with particular focus on its low-latency response to vibrational input signals. Fig. 30 provides an overview of the validation procedure, illustrating the integration of visual stimuli with computer-generated user inputs delivered through the ET’s user interface. These experiments were designed to verify the device’s accuracy and reliability as a sensory substitution tool, as well as to characterize its data acquisition rate.

In addition, we examined the correspondence between motor actuation and distance sensor input to clarify how sensory data are collected, processed, and conveyed to the user. This assessment was critical for establishing the temporal precision of sensorimotor feedback.

To support accurate triggering and synchronization with our EEG system, we incorporated the Brain Products STIMTRAK Acoustical Adapter, selected for its high timing precision. This configuration enabled consistent and reproducible measurements, reinforcing the robustness of the ET’s performance profile [16].

### 7.1 Proximity input latency

To validate the timing of haptic feedback delivered by the motors in response to visual stimuli detected by the LiDAR distance sensor, we quantified the temporal discrepancies associated with feedback delivery. This analysis included both the internal and external motors, as well as the latency between the signal generated by the Teensy microcontroller and the corresponding trigger signal recorded by the STIMTRAK.

Using the internal microphone embedded in the Brain Products STIMTRAK, we precisely detected the onset of motor actuation. The STIMTRAK also delivered a synchronized trigger to the EEG software, allowing direct comparison between the trigger signal generated by the Teensy and the corresponding signal detected by the STIMTRAK.

For this validation, the system operated in binary mode, with an object repeatedly placed in front of and removed from the distance sensor to elicit haptic responses and corresponding ET-generated trigger signals. The STIMTRAK’s microphone was positioned atop the internal motor casing for one set of trials and atop the external motor casing for another, ensuring accurate detection of each motor’s initial vibratory response.

**Fig. 31:**
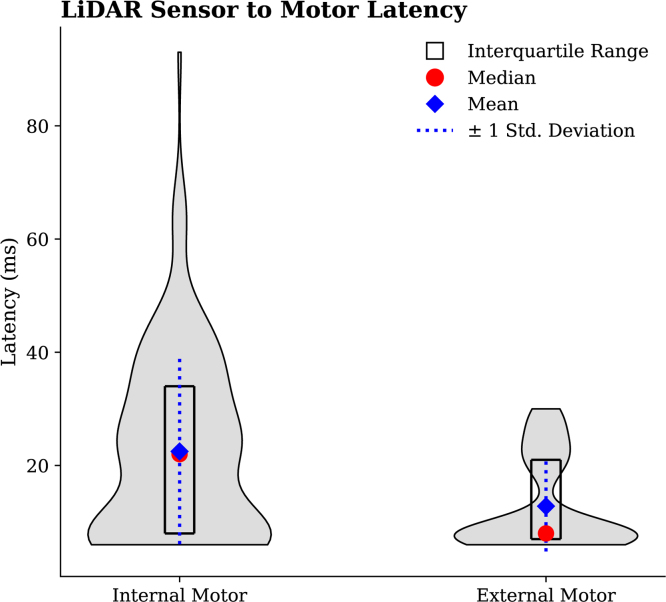
Latency validation between sensor input and actuators (internal and external motor) response.

For the internal motor, 143 samples were recorded, yielding an average latency of 22.48 ms, a median of 22 ms, and a standard deviation of 16.45 ms. For the external motor, 184 samples were collected, resulting in an average latency of 12.81 ms, a median of 8 ms, and a standard deviation of 8.02 ms (see Fig. 31).

### 7.2 Data sampling time

The objective of this analysis was to evaluate the data-logging resolution of the Teensy 4.0 microcontroller under realistic usage conditions and maximal computational load. The ET was configured to operate in binary mode at a resolution of 50 cm, using the binary50 command implemented in the system software.

Distance measurements were collected over a continuous 10-minute interval while maneuvering around the ET. During this period, a total of 58,288 samples were acquired, yielding an effective sampling rate of approximately 97.15 samples per second (97 Hz).

### 7.3 Command-to-actuator latency

This validation test focused on quantifying the latency between command transmission via software (through serial communication) and the resulting motor vibration response. The measurement was conducted using the microphone embedded in the Brain Products STIMTRAK Acoustical Stimulator Adapter, which detected the onset of motor vibration and issued a synchronized trigger signal aligned with the computer-generated command timestamp.

In the experimental protocol, commands were manually sent at predefined intervals to activate the internal motor. The microphone detected the resulting vibrations, and both command and response timestamps were recorded to calculate latency. Specifically, latency was defined as the difference between the software command time and the moment at which the microphone registered motor activation. A parallel procedure was conducted to assess latency in the external motor.

**Fig. 32:**
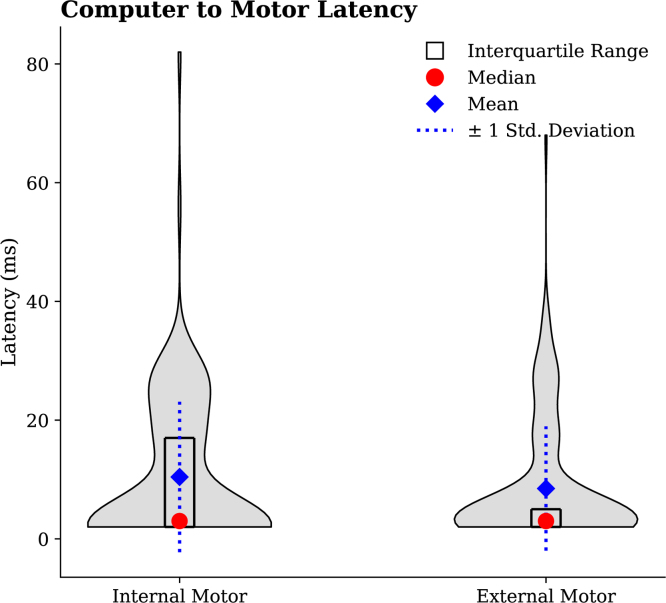
Latency distribution between software command and motor response (internal and external).

Analysis of 121 samples from the internal motor yielded an average latency of 10.42 ms, with a median latency of 3 ms. For the external motor, 149 samples were analyzed, resulting in an average latency of 8.50 ms and a median of 3 ms. The latency distributions for both motors are visualized in the histograms presented in Figs. 32.

**Fig. 33:**
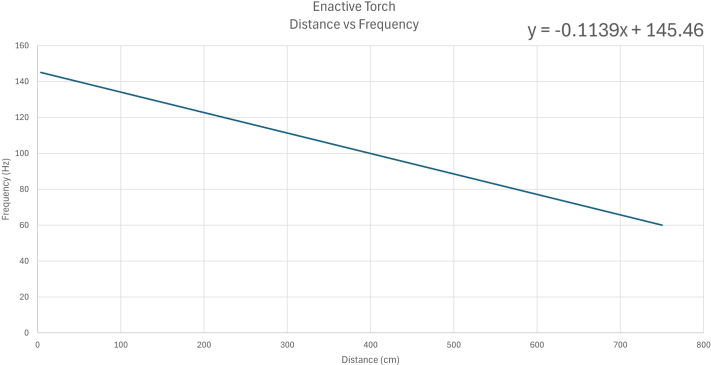
Linear relationship between distance measured by the ET and resulting actuator vibration frequency.

**Fig. 34:**
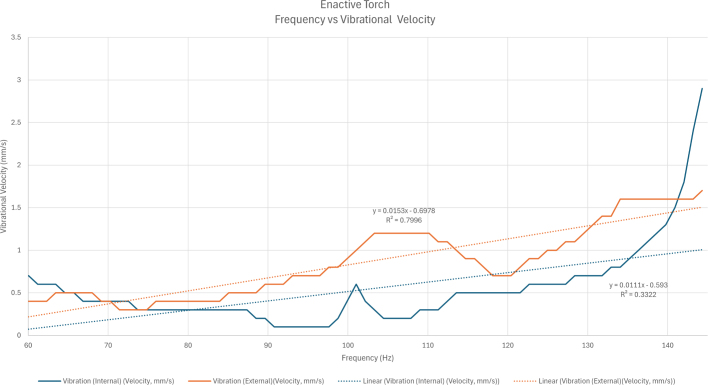
The influence of variations in the actuator frequency on the measured vibrational velocity on the Enactive Torch’s surface.

### 7.4 Distance-to-frequency calibration

In the current configuration, the vibration frequency of the motor is linearly mapped to the distance measured by the LiDAR sensor. The sensor detects objects within a range of 4 cm to 750 cm. When an object is detected at 4 cm, the motor vibrates at 145 Hz. As the distance increases to 750 cm, the vibration frequency linearly decreases to 60 Hz.

This relationship is described by the linear equation:

(1) Y=−0.1139X+145.46

where Y represents the vibration frequency in hertz (Hz) and X is the distance in centimeters (cm). The resulting mapping ensures a smooth and predictable transition in haptic feedback intensity across the full sensing range (see Fig. 33).

**Fig. 35:**
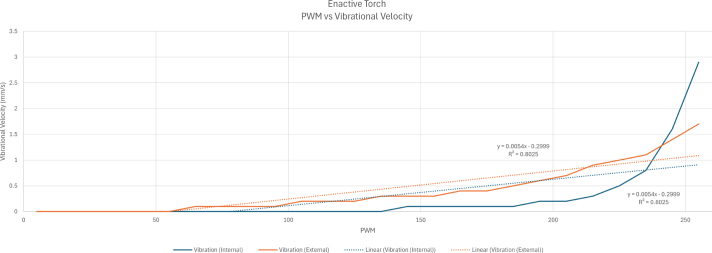
The influence of variations in the PWM signal intensity on the measured vibrational velocity for internal and external motors.

**Fig. 36:**
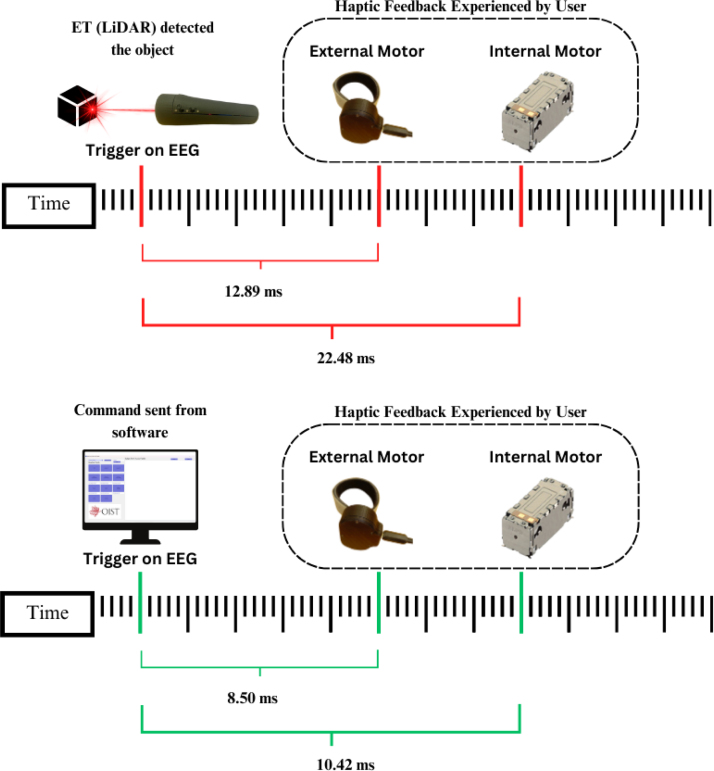
Timeline of the delay for the Enactive Torch motors in relation to the distance sensor and computer software.

### 7.5 Vibrational velocity

This validation experiment quantified the haptic output of the ET by measuring vibrational velocity under two controlled conditions: (1) varying distances, where each distance corresponded to a specific frequency (e.g., 10 cm mapped to a given frequency in Hz), and (2) varying PWM values for both the internal and external motors.

The MT-MotherTool vibration meter (Model VB 8206sd) was used to capture vibration characteristics, including velocity (cm/s, mm/s), acceleration (mm/s${}^{2}$), and displacement (mm). Vibrational velocity, measured in mm/s, was selected as the primary metric due to its precision (rated at 0.1 mm/s) and its sensitivity to subtle changes in haptic output. This ensured reliable, high-resolution data, accurate to one decimal place.

To examine the relationship between distance and vibration, the vibration meter was first calibrated to ensure a stable baseline reading of 0.0 mm/s in the absence of external movement. The sensor was then positioned on the motor, and the distance to an object was manually varied from 10 cm to 750 cm. Vibrational velocity was recorded at each interval, and the results were plotted (see Fig. 34). The same procedure was repeated using the external motor.

For PWM validation, the meter was again calibrated and placed at the typical point of user contact on the motor casing. For the internal motor, PWM values were reduced from 255 to 0 in decrements of 10, and vibrational velocity was recorded at each step. This process was repeated for the external motor. The resulting relationship between PWM values and vibrational velocity is shown in Fig. 35.

Together, these results confirm that the ET system reliably modulates haptic feedback in response to distance inputs, whether generated autonomously through sensor readings or manually through software-defined inputs. To reduce variability and maintain consistency during human subject testing, binary mode was employed for all real-time experimental trials.

### Overall

The validation tests performed on the ET system demonstrated its accuracy across various use cases involving both the external and internal motors. Both the mean and the median were reported due to the median providing information less influenced by outliers. The average sensor-to-motor output delay was approximately 12.89 ms for the external motor and 22.48 ms for the internal motor, suggesting that the device’s casing and internal components may exert a subtle influence on vibrational performance. A similar trend was observed in the computer-to-motor trials, which produced an average delay of 8.50 ms for the external motor and 10.42 ms for the internal motor. Comparatively longer delays were observed in trials involving the LiDAR, likely attributable to the computational overhead and data processing requirements associated with the LiDAR and the Teensy microcontroller (Fig. 36).

Further examination of vibrational velocity disproved the anticipated linear relationship between frequency and haptic feedback. Instead, evaluations of PWM and frequency suggested a relationship more closely resembling an exponential curve, despite notable inconsistencies. Among the irregularities, structural resonance and material damping within the ET hardware likely influenced the motor’s vibrational output, contributing to abrupt peaks in the recorded data. Additionally, the presence of other electrical components within the ET may have altered the motor’s vibratory response, ultimately affecting the user experience. These factors may help explain why the internal and external motors exhibited distinct behaviors, and how the ET can be further improved.

Overall, these findings indicate that the ET device is a viable instrument for laboratory experimentation and holds substantial potential for further refinement. Its demonstrated precision and responsiveness suggest that it can enhance EEG research — particularly in hyperscanning contexts — by integrating sensory substitution mechanisms into existing measurement frameworks.

## CRediT authorship contribution statement

**Stephen Estelle:** Writing – review & editing, Writing – original draft, Visualization, Validation, Software, Methodology, Conceptualization. **Lohith Dayantri:** Writing – review & editing, Writing – original draft, Software, Methodology, Data curation. **Ziwen Meng:** Writing – original draft, Visualization, Software, Data curation. **Brian Morrissey:** Writing – review & editing, Supervision, Project administration. **Tom Froese:** Writing – review & editing, Supervision, Project administration, Funding acquisition.

## Ethics statements

Data acquisition involved human participants. The device and experimental protocol were reviewed and approved by the Human Subjects Research Committee (HSR-2021-004-4) at the Okinawa Institute of Science and Technology Graduate University (OIST).

## Declaration of generative AI and AI-assisted technologies in the manuscript preparation process

During the preparation of this work the authors used ChatGPT in order to provide additional support in data analysis and editing. After using this tool/service, the authors reviewed and edited the content as needed and take full responsibility for the content of the published article.

## Funding

This work was supported by the 10.13039/501100004199Okinawa Institute of Science and Technology Graduate University, Japan (OIST).

## Declaration of competing interest

The authors declare that they have no known competing financial interests or personal relationships that could have appeared to influence the work reported in this paper.
